# Detection of reactive oxygen metabolites in malignant and adjacent normal tissues of patients with lung cancer

**DOI:** 10.1186/1477-7819-11-9

**Published:** 2013-01-17

**Authors:** Hacer Kuzu Okur, Meral Yuksel, Tunc Lacin, Volkan Baysungur, Erdal Okur

**Affiliations:** 1 Department of Pulmonology, Sureyyapasa Chest Disease and Thoracic Surgery Training and Research Hospital, Basibuyuk-Maltepe, Istanbul, 34726, Turkey; 2 Vocational School of Health Related Professions, Department of Medical Laboratory, Marmara University, Haydarpasa Campus, Istanbul, 34668, Turkey; 3 Department of Thoracic Surgery, Sureyyapasa Chest Disease and Thoracic Surgery Training and Research Hospital, Basibuyuk-Maltepe, Istanbul, 34726, Turkey; 4 Department of Thoracic Surgery, Acibadem University Medical School, Istanbul, Gulsuyu_Maltepe, Istanbul, 34726, Turkey

**Keywords:** Reactive oxygen metabolites, Lung cancer, Oxidative stress

## Abstract

**Background:**

Different types of reactive oxygen metabolites (ROMs) are known to be involved in carcinogenesis. Several studies have emphasized the formation of ROMs in ischemic tissues and in cases of inflammation. The increased amounts of ROMs in tumor tissues can either be because of their causative effects or because they are produced by the tumor itself. Our study aimed to investigate and compare the levels of ROMs in tumor tissue and adjacent lung parenchyma obtained from patients with lung cancer.

**Methods:**

Fifteen patients (all male, mean age 63.6 ± 9 years) with non-small cell lung cancer were enrolled in the study. All patients were smokers. Of the patients with lung cancer, twelve had epidermoid carcinoma and three had adenocarcinoma. During anatomical resection of the lung, tumor tissue and macroscopically adjacent healthy lung parenchyma (control) that was 5 cm away from the tumor were obtained. The tissues were freshly frozen and stored at −20°C. The generation of ROMs was monitored using luminol- and lucigenin-enhanced chemiluminescence (CL) techniques.

**Results:**

Both luminol (specific for ^.^OH, H_2_O_2_, and HOCl^-^) and lucigenin (selective for O_2_^.-^) CL measurements were significantly higher in tumor tissues than in control tissues (*P* <0.001). Luminol and lucigenin CL measurements were 1.93 ± 0.71 and 2.5 ± 0.84 times brighter, respectively, in tumor tissues than in the adjacent parenchyma (*P* = 0.07).

**Conclusion:**

In patients with lung cancer, all ROM levels were increased in tumor tissues when compared with the adjacent lung tissue. Because the increase in lucigenin concentration, which is due to tissue ischemia, is higher than the increase in luminol, which is directly related to the presence and severity of inflammation, ischemia may be more important than inflammation for tumor development in patients with lung cancer.

## Background

Lung cancer, which is the most common cause of cancer deaths worldwide among both men and women, accounts for 28% of cancer deaths and approximately 6% of all deaths [1]. Tobacco smoke is the main cause of lung cancer and is responsible for 87% of all lung cancers in the United States [1,2]. The risk increases with the amount of tobacco used and the length of time for which it has been used. Cigarette smoke, a major source of exogenous oxidants, leads to chronic airway inflammation with accumulation and activation of leucocytes, which produce high levels of reactive oxygen metabolites (ROMs) and nitric oxide [3,4]. Lung tissue is protected against these oxidants by a variety of antioxidant mechanisms such as superoxide dismutases. An imbalance between oxidant and antioxidant levels induces oxidative stress in lung tissues [5]; thus, excessive and inappropriate production of endogenous and/or exogenous reactive oxygen species and nitric oxide is implicated in the pathogenesis of lung cancer [1,6,7]. Free radicals are well-known mutagenic agents and cause genotypic changes that may lead to the development of cancer [8].

Although it is difficult to quantitate ROMs because of their short-lived and reactive nature, the chemiluminescence (CL) method used in the present study is a simple and reproducible technique. The two CL probes, luminol and lucigenin, differ in their selectivity. Lucigenin is particularly sensitive to the superoxide radical (O_2_^.-^), whereas luminol detects hydrogen peroxide (H_2_O_2_), hydroxyl radicals (^.^OH), hypochlorite (ClO^-^), peroxynitrite (ONOO^-^), and lipid peroxyl radicals [9].

Detection of high levels of ROMs, which are known to be increased by ischemia or inflammation, in tumor tissues may be attributable either to their role in the etiology of cancer or to the presence of the tumor itself. The present study was designed to explore the intensity of oxidative stress in patients with lung cancer by assessing the generation of ROMs in tumor and normal lung parenchyma and comparing the levels of metabolites in both tissues.

## Methods

### Patients

Fifteen consecutive patients with non-small cell lung carcinoma who were operated on in our clinic were included in the study. All patients were smokers and had no preoperative neoadjuvant treatment and no previous history of carcinoma of any type. Patients with tumors less than 2 cm in size or endobronchial tumors were not included because obtaining a 1-cm sample from such a small tumor might disturb the pathological examination. After routine preoperative evaluation and staging, the patients underwent anatomical lung resection (either lobectomy or pneumonectomy) together with mediastinal lymph node dissection. Informed consent of the patients and approval of the ethics committee of Marmara University Medical School were obtained.

### Experimental design

Immediately after completion of lung resection and removal of the surgical specimen, two tissue samples were obtained from each patient: the first sample was obtained from the lung tumor and the second was obtained from adjacent lung parenchyma (for the control group) at least 5 cm away from the gross margin of the lung tumor. The tissues were then washed with saline (0.9% NaCl) to remove blood, freshly frozen, and stored at −20°C until they were examined.

### Chemiluminescence measurement

To assess the role of ROMs in this study, CL derived from luminol and lucigenin was measured as an indicator of radical formation. CL measurements are based on light emission with specific enhancers such as luminol, lucigenin, luciferase, and horseradish peroxidase. The benefits of this method include rapidity, ultrasensitive detection limits, and broad applicability. In clinical research, the sensitivity of this method has led to its use in a wide range of applications, especially those that involve monitoring of reactive oxygen species [10].

In our experiment, tissues were thawed and washed with saline. Luminescence of the tissue samples was recorded at room temperature using a Junior LB 9509 luminometer (EG&G Berthold, Bad Wildbad, Germany) in the presence of enhancers. Tissue specimens were cut into two pieces and placed into tubes containing PBS-HEPES buffer (0.5 mol/L phosphate buffered saline containing 20 mmol/L HEPES, pH 7.2). ROMs were quantitated after addition of the enhancer (lucigenin or luminol) to a final concentration of 0.2 mmol/L. After the measurements were made, the tissues were removed from the tubes, dried on filter papers, and weighed. All chemiluminometric counts were obtained at 1-min intervals for 5 min, and the results were expressed as areas under the curve (AUCs) of relative light units (rlu) for 5 min per mg of tissue [9]. The calculation was based on the integration of the curve using the trapezoidal rule (a linear approximation).

### Statistical analysis

The various degrees of oxidative stress in different types of lung tumors, including epidermoid carcinoma and adenocarcinoma, and adjacent lung tissues were statistically analyzed. All data are expressed as means ± SEM. Groups of data were compared using a paired *t* test. The results were considered significant when the *P* value was less than 0.05. Calculations were performed using GraphPad Prism 3.0 (GraphPad Software, San Diego, CA, USA).

## Results and discussion

We examined a total of 15 patients, all of whom were male with a mean age of 63.6 ± 9 years (range 48 to 74 years). Patients’ characteristics are summarized in Table 1. 

**Table 1:** Characteristics of patients

	**Age (years)**	**Locations**	**TNM**	**Histological type**
1	59	RUL	T_2_N_0_M_0_	Adeno ca
2	73	RUL	T_1_N_1_M_0_	Epid ca
3	71	LLL	T_1_N_1_M_0_	Epid ca
4	65	RUL	T_1_N_0_M_0_	Epid ca
5	58	RUL	T_1_N_1_M_0_	Epid ca
6	65	RUL	T_2_N_1_M_0_	Adeno ca
7	60	RLL	T_1_N_1_M_0_	Epid ca
8	74	RML	T_2_N_0_M_0_	Epid ca
9	49	LLL	T_2_N_1_M_0_	Adeno ca
10	72	LLL	T_1_N_1_M_0_	Epid ca
11	78	LUL	T_2_N_0_M_0_	Epid ca
12	53	LLL	T_2_N_0_M_0_	Epid ca
13	67	LUL	T_2_N_1_M_0_	Epid ca
14	62	RUL	T_2_N_0_M_0_	Epid ca
15	48	LLL	T_1_N_1_M_0_	Epid ca

Adeno ca, adeno carcinoma; Epid ca, epidermoid carcinoma; LLL, left lower lobe; LUL, left upper lobe; RLL, right lower lobe; RML, right medial lobe; RUL, right upper lobe; TNM, tumor, node, metastasis (classification system).

**Figure 1:**
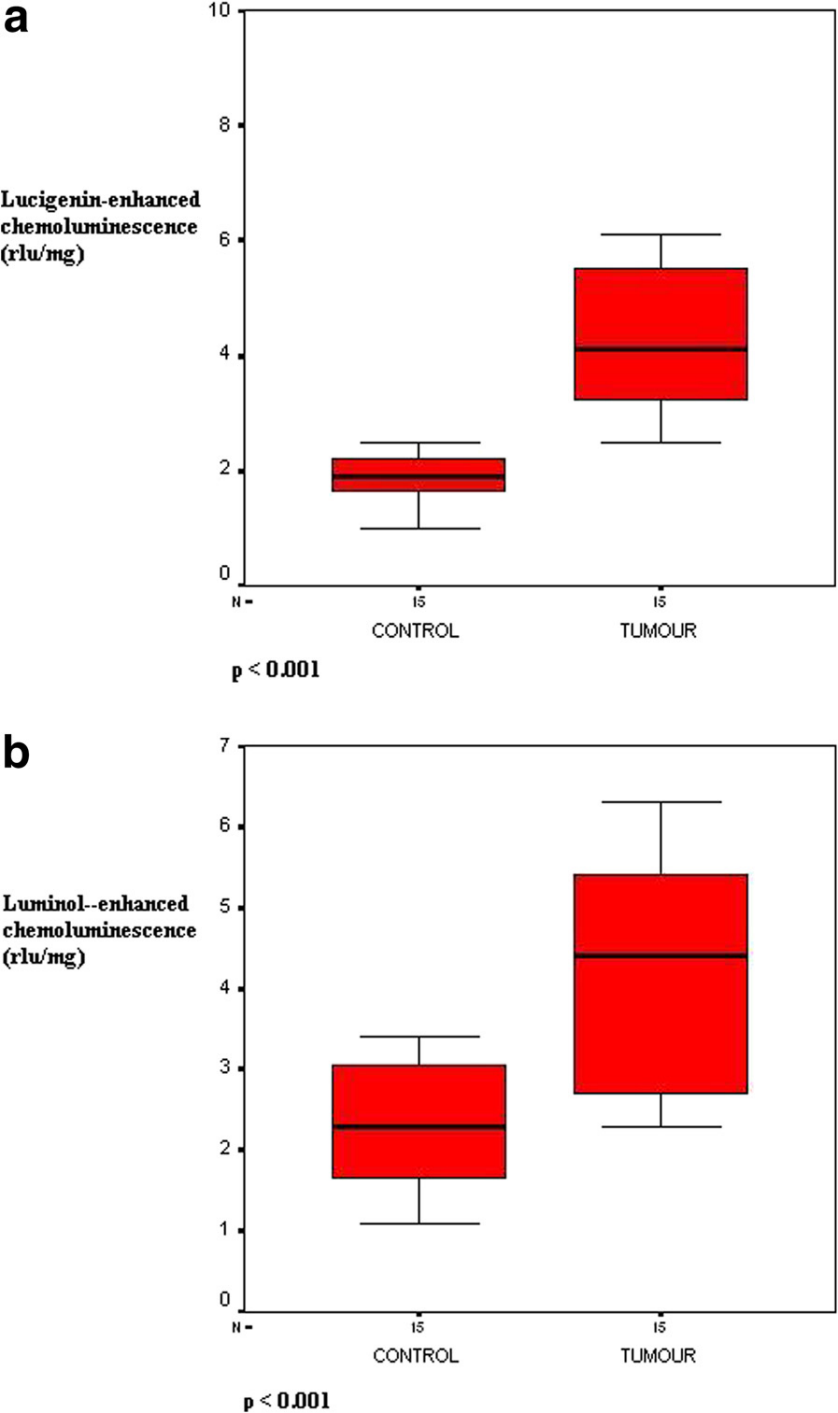
**Lucigenin (a) and luminol (b) chemiluminescence (CL) levels in the control and tumor groups.** Rlu, relative light units.

In all patients, the lucigenin and luminol CL values were higher in cancerous lung tissues than in adjacent lung tissues. The luminol CL level in tumor tissues was significantly higher than that in control tissues (4.32 ± 0.38 rlu/mg tissue vs. 2.26 ± 0.22 rlu/mg tissue, *P* <0.001) (Figure 1a). Similarly, the lucigenin CL level showed a marked increase in the tumor tissues as compared to control tissues (4.49 ± 0.44 rlu/mg tissue vs. 1.91 ± 0.12 rlu/mg tissue, *P* <0.001) (Figure 1b). 

In this study, we investigated the role of oxidative stress in lung cancer. We demonstrated that ROM levels, as measured by the CL method, were significantly higher in the cancerous tissues of lung cancer patients than in control tissues obtained from the same patients.

The antitumor defense mechanisms of the body result in continuous oxidative stress and inflammatory responses, which contribute to the various types of oxidative damage observed in lung cancer [11]. It may be hypothesized that chronic injury to the epithelium caused by the production of ROMs may lead to lung cancer. Free radicals that play a role in carcinogenesis may originate from cigarette smoke, air pollution, or activated phagocytes, the latter of which are a consequence of chronic inflammatory diseases [12].

In a report that examined lipid peroxidation in whole blood samples obtained from patients with lung cancer, the authors reported that the level of malondialdehyde (MDA, a lipid peroxidation product) in patients with early-stage (stage I and II) lung cancer was not significantly altered compared to the control group [7]. However, in patients with advanced-stage lung cancer, the MDA level was significantly higher. Additionally, the level of whole blood glutathione (an intracellular antioxidant) was decreased in patients with both early- and advanced-stage lung cancer. In another study, the ROM levels in serum samples from patients with small cell carcinoma, adenocarcinoma, and epidermoid carcinoma were higher than the levels detected in control subjects [13]. Zieba *et al*. reported that lipid peroxidation was higher in cancerous tissue samples than in normal lung parenchyma and demonstrated that thiobarbituric acid-reactive substances, which are markers of lipid peroxidation, were positively correlated with both clinical stage and spontaneous generation of H_2_O_2_ in tumor tissue [4].

In our study, luminol-enhanced CL measurements were significantly higher in tumor specimens than in adjacent pulmonary parenchyma tissues. Our method, which is based on the measurement of spontaneously released ROMs, is also capable of measuring H_2_O_2_ and another substances such as hydroxyl radicals (^.^OH), hypochlorite ions (ClO^-^), peroxynitrite ions (ONOO-), and lipid peroxyl radicals. Our results show that H_2_O_2_ and other compounds are released from lung tumor specimens. In an animal study, rats were exposed to cigarette smoke, and the luminol-enhanced CL technique was used to measure ROMs in lung and laryngeal tissues [14]. The results indicated that cigarette smoking increased luminol-enhanced CL measurements, and vitamin E decreased ROM release significantly. H_2_O_2_ can be converted to ^.^OH in the presence of metal ions via the Fenton reaction or by neutrophil infiltration; alternatively, it can be converted to HOCl^-^ in the presence of chloride ions via the myeloperoxidase enzyme. In our study, luminol-enhanced CL amplification can also be used to measure ^.^OH and HOCl^-^. HOCl^-^ is usually produced by polymorphonuclear leukocytes at an inflammation site. In a group of patients with non-small cell lung cancer, myeloperoxidase activity was lower and glutathione levels were higher in patients with malignant disease [15]. Reports of increased inflammatory markers and C-reactive protein [16] as well as an acute phase response in cancer patients indicate that both systemic and local (that is, at the tumor site) inflammatory reactions are closely associated with the presence of a tumor [4]. In addition, some studies indicate that the tumor tissue itself could be the source of ROMs and lipid peroxidation products [17].

Ischemia is the major cause of superoxide generation in cancer tissues. Ischemia leads to glucose and adenosine triphosphate (ATP) depletion, which in turn leads to a decrease in sodium-potassium ATPase (Na-K ATPase) activity and causes depolarization of the cells. In these depolarized cells, intracellular calcium increases, resulting in activation of the xanthine-hypoxanthine system and production of superoxide radicals. Superoxide radicals, which are highly reactive, damage membrane phospholipids, cellular proteins, and DNA [18]. In our study, the levels of lucigenin-enhanced CL were higher in tumor samples than in parenchymal tissues. Formation of superoxide radicals can be prevented via superoxide dismutase (SOD) enzymes. In an immunohistochemical study, the expression levels of both Mn-SOD (mitochondrial form) and Cu/Zn-SOD (cytoplasmic or nuclear form) in the lungs of patients with squamous cell carcinoma were significantly lower than the expression levels of these enzymes in luminal cells and uninvolved epithelium of the same patients [19]. These results demonstrate that intracellular and intramitochondrial SODs are not capable of reducing superoxide radical generation, and our study also provided evidence to support this finding based on lucigenin-enhanced CL measurements.

The monitoring of ROM generation in freshly frozen tissue samples using the CL technique has been described in two studies by Ohoi *et al*. [20,21]. Cicenas *et al*. and Toklu *et al*. also used this method for freshly frozen tissue samples of breast and colon cancers as well as for urogenital tissues [9,22,23]. Our study is the first to measure ROM levels in lung cancer tissue samples.

A limitation of our study is the small sample size (15 patients). This was a small pilot study. Similar studies with larger sample size, at least 100 patients, are needed to confirm our results.

## Conclusion

In general, evidence suggests that lucigenin-enhanced superoxide radical generation is increased by ischemia in tissues, whereas the luminol level is increased in the presence of inflammation and is directly related to inflammatory severity. In our study, the mean concentrations of lucigenin and luminol in cancerous lung tissue were significantly higher than their concentrations in adjacent lung parenchyma. A higher increase in the concentration of lucigenin compared with luminol shows that tissue ischemia may be more important than inflammation for tumor development in lung cancer patients.

## Competing interests

All the authors declare that they have no competing interests.

## Authors’ contributions

HKO and MY designed the research, TL, VB and EO performed the operations and collected the tissue samples. MY perfomed the laboratory measurements and analyses. HKO and EO analysed the data. HKO, MY and EO wrote the paper. All authors read and approved the final manuscript.
